# Application and Comparison of FPGA-Based Carry Chain TDC and DDMTD Schemes in High-Precision Time Synchronization

**DOI:** 10.3390/s26031052

**Published:** 2026-02-05

**Authors:** Yuzhen Huang, Jiajie Yu, Wenlong Xia, Qinggong Guo, Linyu Huang

**Affiliations:** College of Electronics and Information Engineering, Sichuan University, No. 24, Section 1, Yihuan Road, Wuhou District, Chengdu 610065, China; 2023222055139@stu.scu.edu.cn (Y.H.); 2023222055128@stu.scu.edu.cn (J.Y.); guoqingong@scu.edu.cn (Q.G.); lyhuang@scu.edu.cn (L.H.)

**Keywords:** field programmable gate array (FPGA), time synchronization, phase difference measurement, time to digital converter (TDC), digital dual mixer time difference (DDMTD), dynamic phase shifting

## Abstract

High-precision phase difference measurement based on field-programmable gate arrays (FPGA) has important application requirements in fields such as high-stability time-frequency transmission, signal synchronization, and precision testing. Addressing the limitations of traditional methods in terms of temperature stability and measurement accuracy, this paper proposes two high-precision phase difference measurement schemes based on the FPGA platform. An eight-parallel-multi-carry chain time-to-digital converter (TDC) and digital dual-mixer time difference (DDMTD) measurement modules are constructed to perform high-precision phase difference measurements on the phase-shifted output signal of the MMCM dynamic phase-shifted module. Results show that at room temperature (25 °C), the single-carry chain TDC exhibits better measurement accuracy than the DDMTD, and the single-carry chain TDC’s measurement error range of 4.7–6.0 ps is superior to the DDMTD’s 20–75 ps error range. Under different temperature conditions, the eight-parallel-multi-carry chain TDC consistently demonstrates superior measurement accuracy, resolution, and temperature stability compared to the single-carry chain TDC. In terms of measurement accuracy, under room temperature conditions, in three sets of phase difference tests (178.5714 ps, 357.1428 ps, and 535.7142 ps), the measurement error of the eight-parallel-multi-carry chain TDC was controlled within 4.6 ps, which is better than the 4.7–6.0 ps error range of the single-carry chain TDC. Average resolution: The average resolution of the single-carry chain TDC was 6.329 ps, while the average resolution of the eight-parallel-multi-carry chain TDC improved to 0.833 ps. Temperature stability: Within the temperature range of 10 °C to 100 °C, the temperature coefficient of the single-carry chain TDC was 0.002127 ps/°C, while the temperature coefficient of the eight-parallel-multi-carry chain TDC decreased to 0.000564 ps/°C. This paper also summarizes the advantages and limitations of the above methods in terms of implementation complexity and robustness, providing a reference for the optimized design of high-precision phase difference measurement technology for FPGA platforms.

## 1. Introduction

Time synchronization plays a critical role in high-precision measurement systems [1,2], satellite navigation [3], communication networks [4], and distributed architectures [5], where timing accuracy directly impacts overall system performance and reliability. With the continuous evolution of modern electronic systems toward higher data rates and tighter clock coordination, sub-nanosecond and even picosecond-level time synchronization has become a fundamental requirement in many emerging applications [6,7,8]. For instance, in global navigation satellite systems (GNSS), a timing error of only one nanosecond can result in positioning inaccuracies on the order of several tens of centimeters [9]. Similarly, in high-speed communication and distributed computing systems, clock misalignments may lead to link instability, data corruption, or system-level performance degradation [10]. Consequently, the development of hardware-feasible time synchronization techniques that simultaneously achieve high resolution and long-term stability remains a problem of both theoretical and practical importance.

Traditional time measurement methods typically rely on high-frequency counters or dedicated time-to-digital converters (TDCs). Counter-based methods are limited by the reference clock period and typically provide resolutions from 100 ps to a few nanoseconds, which leads to increased power consumption but relatively low area overhead. Time-domain TDC architectures, such as vernier delay lines and DLL-based architectures, can improve resolutions to approximately 5–50 ps, but at the cost of additional delay components, calibration circuitry, and high sensitivity to process, voltage, and temperature (PVT) variations, resulting in moderate area and power overhead. Voltage-domain TDCs based on high-resolution analog-to-digital converters (ADCs) can achieve resolutions below 10 ps, but suffer from excessive power consumption and large chip footprints, limiting their large-scale integration capabilities [11,12,13,14,15]. Although these methods can theoretically provide fine time resolution, their hardware complexity, power consumption costs, and limited scalability hinder their widespread application in complex systems [16]. In recent years, with the rapid development of FPGA platforms in terms of structural flexibility, parallel processing capabilities, and abundant on-chip clock resources, FPGA-based time measurement technology has become an important research direction. In particular, the sub-nanosecond delay characteristics of the FPGA carry chain structure and the fine phase control provided by on-chip clock management modules (such as MMCM/PLL) enable high-resolution timing measurements without relying on custom ASIC designs [17]. This approach offers significant advantages in terms of cost-effectiveness, configurability, and scalability, making FPGA-based TDCs attractive for a wide range of applications [18,19,20].

Among existing FPGA-based TDC techniques, carry chain TDC and DDMTD represent two representative and complementary solutions. Carry chain TDC exploits the intrinsic propagation delay of FPGA carry logic to form a fine-grained delay line, with timing information extracted by encoding the signal transition position along the chain [21,22]. To further improve resolution, advanced schemes such as wave union TDC and multi-edge sampling have been proposed, which effectively interpolate multiple transitions within a single measurement cycle [23]. Additionally, bin-by-bin calibration techniques, typically based on code-density tests, are widely employed to compensate for nonuniform bin widths and reduce differential and integral nonlinearity. While these methods can significantly enhance resolution, they often introduce increased hardware complexity, higher resource consumption, and substantial calibration overhead, particularly when extended to large-scale or multi-channel systems. In contrast, the DDMTD technique estimates time differences by generating a low-frequency beat signal through digital mixing and phase detection, offering excellent suppression of noise, frequency offset, and long-term drift [24]. As a result, DDMTD is commonly adopted in high-stability clock comparison and long-term synchronization applications. However, its performance is highly dependent on reference clock quality, and its inherent low-bandwidth nature limits real-time responsiveness.

Despite the substantial progress in FPGA-based TDC architectures, several practical challenges remain. Carry chain TDC implementations are highly sensitive to PVT variations, which induce nonlinear and nonstationary delay behavior in the carry logic. In industrial environments spanning wide temperature ranges (e.g., −40 °C to 85 °C), such variations can significantly degrade timing resolution, increasing differential nonlinearity and causing effective resolution to deteriorate from the picosecond level to several tens of picoseconds [25,26]. Moreover, while sophisticated calibration methods can mitigate these effects, they often require frequent recalibration or increased logic and memory resources. Similarly, although MMCM-based dynamic phase shifting provides fine digital phase control, its effective accuracy is constrained by finite phase step resolution, jitter, and on-chip noise, leading to discrepancies between theoretical and measured phase offsets [27].

Motivated by these limitations, this work proposes and systematically evaluates a parallel multi-carry chain TDC architecture based on a partitioned structure, aiming to balance timing resolution, robustness, and hardware efficiency. Unlike wave-union or heavily oversampled designs that prioritize resolution at the cost of resource utilization, the proposed multi-carry chain partitioning approach improves effective timing precision and temperature robustness through spatial averaging and parallelism, while maintaining moderate logic overhead. By integrating MMCM dynamic phase shifting, carry chain TDC, and DDMTD phase measurement within a unified FPGA-based platform, this study enables a comprehensive comparison of accuracy, temperature drift characteristics, and implementation complexity under identical operating conditions.

The main contributions of this paper are summarized as follows:

1. A unified FPGA-based time synchronization platform integrating MMCM dynamic phase shifting, carry chain TDC, and DDMTD phase measurement is developed.
2. A temperature-controlled experimental framework is established to systematically investigate the impact of temperature variation on both single-chain and parallel multi-carry chain TDC architectures.
3. The proposed parallel multi-carry chain TDC is comprehensively evaluated in terms of measurement accuracy, temperature-induced drift, and hardware cost, and its performance trade-offs are analyzed in comparison with existing FPGA-based TDC techniques, providing practical guidance for the design of high-precision and high-stability time synchronization systems.

## 2. Experimental Methods and System Design

### 2.1. Overall Experimental Design

**Figure 1:**
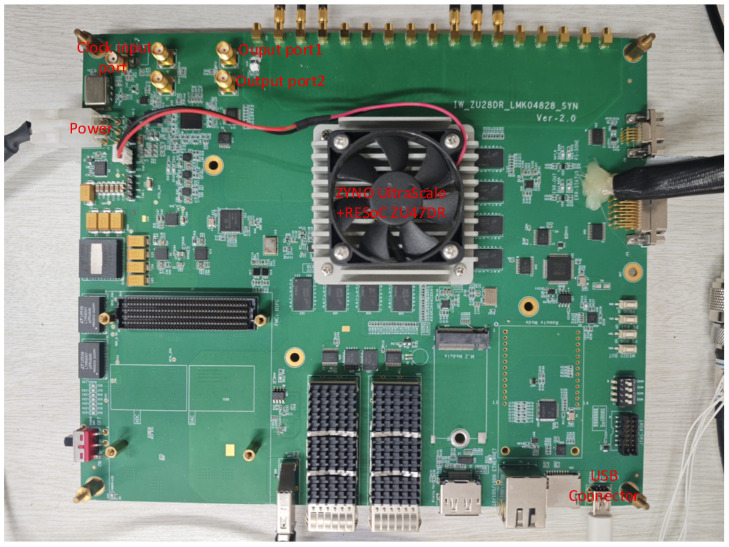
A picture of the evaluation board.

The core hardware platform selected for this experiment is the Xilinx 47DR FPGA development board. The development board is equipped with a medium-high density FPGA chip based on the Ultrascale series architecture and has very rich hardware resources: it integrates about 400,000 logic units (LUTs) and 850,000 flip-flops (FFs), which is sufficient to ensure the stable implementation of complex logic circuits. In addition, it is equipped with 60 MB of on-chip block RAM (BRAM), which provides reliable support for high-speed caching and interaction with experimental data, and has sufficient carry resources, which can fully meet the core requirements of this experiment to build a large-scale carry chain delay line. It is with the above-mentioned complete hardware resources that a solid hardware foundation is laid for the smooth progression of subsequent experiments. In terms of the clock system, a high-precision, low-jitter 10 MHz rubidium atomic clock is used to provide clock resources and supports the MMCM/PLL clock management unit inside the FPGA. The STM-Rb-N rubidium atomic clock (Tongxiang Technology Co., Ltd., Beijing, China) is a clock characterized by excellent short-term frequency stability, small size, light weight, and low power consumption. This rubidium clock can output a stable 10 MHz frequency signal, and its frequency accuracy is better than 5 × 10^−11^ after approximately 300 s of being powered on. Its single-sideband phase noise is better than −155 dBc/Hz and −158 dBc/Hz at frequency offsets of 1 kHz and 10 kHz, respectively. These specifications indicate that the clock has extremely low phase noise levels across both near-end and far-end frequency offsets. The digital dynamic phase-shift function of the MMCM supports picosecond-level step accuracy and can generate multiple sets of highly stable phase-shift test clocks, which is an important basis for the phase-shift experiment in this study [28]. In addition, the integrated XADC power and temperature monitoring module on the development board can record the FPGA’s operating temperature in real time, facilitating the analysis of the impact of temperature changes on delay chain stability and measurement errors. In the current configuration, the XADC’s internal ADC clock is 5 MHz, and the total conversion rate is 200 kSPS; with 256 averaging operations enabled, the effective output rate per channel is approximately 781 SPS. With a resolution of 10 bits and using an external reference voltage of 1.25 V and an external reference voltage tolerance of ±0.2%, the ADC sampling accuracy may be affected by ±2 LSB (10 bits). Figure 1 shows a photograph of our evaluation board. The USB interface is used to send results to a computer. The two SMA ports, output1 and output2, are used to output two phase-shifted signals containing the phase difference.

The entire experimental system consists of three main functional modules:

(1) Signal generation and reference measurement module: This module uses an MMCM dynamic phase-shifting module to generate three sets of signals with phase differences, and measures the phase difference by using a Tektronix DPO71254C digital phosphor oscilloscope (Tektronix, Inc., Beaverton, OR, USA) as a reference value. This oscilloscope has a bandwidth of up to 23 GHz and a real-time sampling rate of up to 50 GS/s. For recording lengths of less than 10 μs, its time base stability is better than 1.0 ps *RMS*.
(2) Phase measurement module: This module includes a single-carry chain TDC, an 8-parallel-multi-carry chain TDC, and a DDMTD digital time difference measurement circuit; all the above are used to compare the accuracy and stability of different measurement methods.
(3) Data acquisition and analysis module: This module consists of an FPGA-internal integrated logic analyzer (ILA) and a host computer responsible for acquiring raw data and visualizing measurement results.

Through these experiments, this study enables a horizontal comparison of different measurement methods in a fully controllable testing environment, providing a practical and effective experimental basis for the design of a high-precision time synchronization system based on an FPGA.

### 2.2. MMCM Phase-Shifting Module

**Figure 2:**
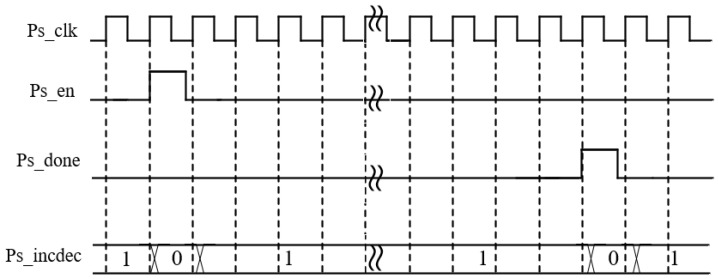
MMCM phase-shift process waveform diagram.

The MMCM module inside the Xilinx 47DR is responsible for outputting two clock signals with the same frequency but an adjustable phase difference. The minimum phase resolution for phase adjustment depends on the VCO frequency, which is 1000 MHz in MMCM. Its implementation principle is shown in Figure 2. Ps_clk is the driving clock for the phase-shift control signal. Ps_en is the signal that enables us to control the phase-shift; a high Ps_en pulse shifts the output phase by 1/56 of a VCO cycle [29,30]. Ps_incdec is the signal used for positive and negative phase shifts; 1 indicates a positive shift, and 0 indicates a negative shift. Ps_done is the phase shift completion flag signal, which is a high level of one Ps_clk signal; the next Ps_en signal only takes effect once this flag has been raised to a high level. The Ps_clk clock frequency used in this paper is 200 MHz.

In this experiment, by adjusting the value of the phase register ps_value, three sets of test signals with phase differences were generated. The relationship between ps_value and the phase-shift value Δt is shown in Equation (1).

(1) $$\Delta t=ps\_value\times \frac{1}{56}\times \frac{1}{f_{VCO}}$$

We set ps_value to 10, 20, and 30, respectively, generating three sets of test signals with theoretical phase differences of 178.5714 ps, 357.1428 ps, and 535.7142 ps.

Due to the digital interpolation and noise characteristics within the MMCM, its output phase difference is not ideal, exhibiting slight deviations and jitters. To obtain the true phase difference for subsequent measurement reference, this experiment used a high-sampling-rate oscilloscope to measure the phase of the phase-shifted signal. The testing signal was input into channels 3 and 4 of the oscilloscope, and the average and variance were read; details will not be elaborated further.

### 2.3. DDMTD

#### 2.3.1. DDMTD Implementation Principle

**Figure 3:**
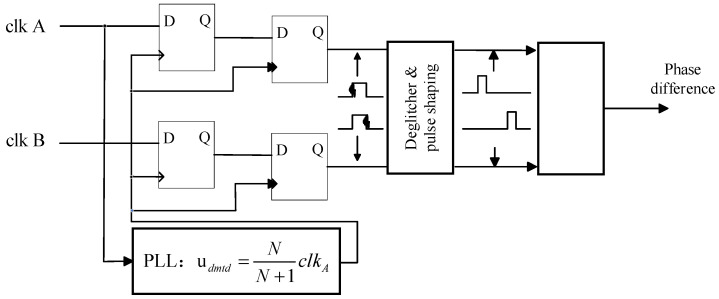
Block diagram of DDMTD.

Figure 3 shows the design principle of a full DDMTD implemented based on FPGA. First, the metastability of the two clock signals being tested, clk_A and clk_B, is removed. clk_A and clk_B are two signals with the same frequency but a phase difference. Then, an auxiliary clock $u_{dmtd}$ is generated by the FPGA’s MMCM to mix and sample the two clock signals being tested. When the mixing clock is very close to the frequency of the clock under testing, the output clock after mixing needs to be de-glitched to obtain a stable state. Then, the phase difference between the two output clocks is counted by a counter to calculate the phase difference of the clock signal under testing. The frequency relationship can be shown as Equation (2):

(2) $$u_{dmtd}=\frac{N}{N+1}clk\_A$$

where $u_{dmtd}$ is the auxiliary clock, N>0, and clk_A is the clock to be measured. It is easy to see that there is an amplification factor N between the auxiliary clock and the clock to be measured. The selection of this value is crucial. Theoretically, N should be made as large as possible because the size of N directly affects the phase resolution ${△t}_{min}$ of DDMTD in Equation (3).

(3) $${△t}_{min}=\frac{1}{\left[N\cdot {clk}_{A}\right]}$$

Generally speaking, a higher N value can achieve a greater resolution. However, in actual hardware systems, increasing this parameter is often strictly constrained by hardware clock resources, making it difficult to configure a larger N value. This creates a technical trade-off between resolution improvement and hardware resource constraints.

**Figure 4:**
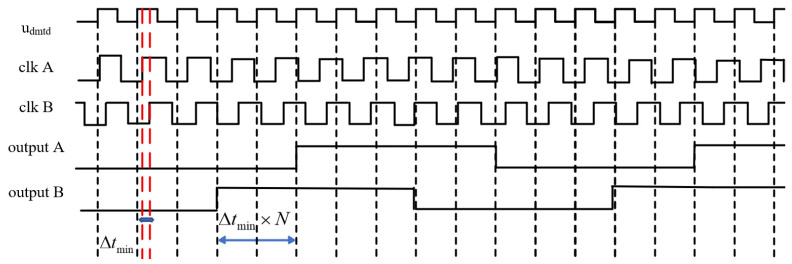
The $u_{dmtd}$ signal is mixed with the clock signals clk_A and clk_B to generate two low-frequency extended beat signals, which are then processed to accurately calculate the phase shift. The arrows represent that time interval, indicating the phase difference between the two signals and the phase difference after being amplified by N times.

Inside the FPGA, the $u_{dmtd}$ clock is used to mix and sample the clock signals clk_A and clk_B under test in Figure 4. The D flip-flop outputs the mixed low-frequency clock signals output_A and output_B, meaning the phase difference between clk_A and clk_B is linearly amplified by a factor of N after mixing. Therefore, by measuring the phase difference between output_A and output_B and applying a linear relationship, the phase difference between clk_A and clk_B can be calculated.

#### 2.3.2. DDMTD Phase Difference Measurement

The phase difference Δt between the tested clock signals clk_A and clk_B is calculated in Equation (4).

(4) $$\Delta t={(\Delta t}_{beat})\left[1-\left(\frac{N}{N+1}\right)\right]=\frac{{delta}_{dmtd}}{clk\_cnt\times N}$$

where N is the amplification factor, and ${\Delta t}_{beat}$ can be obtained from the number of cycles of the auxiliary clock $u_{dmtd}$. For example, in Figure 4, ${\Delta t}_{beat}$ contains two $u_{dmtd}$ auxiliary clock cycles, so ${\Delta t}_{beat}=\frac{2}{f\left(u_{dmtd}\right)}$, clk_cnt is the sampling clock, used to calculate the time width of ${\Delta t}_{beat}$. ${delta}_{dmtd}$ is the number of clk_cnt clock cycles contained in ${\Delta t}_{beat}$, so the phase difference can be calculated in Equation (4). The relevant parameters of DDMTD in this paper are set as follows, which will not be repeated later. The two clocks under test, clk_A and clk_B are both 200 MHz clocks, while the $u_{dmtd}$ auxiliary clock is 204.167 MHz. The amplification factor can be calculated to be 47.9961, and the sampling clock is 500 MHz.

### 2.4. Single-Carry Chain TDC

While using a clock counter alone for time interval measurement can cover a large measurement range, the DDMTD method is limited by the stability of the onboard crystal oscillator of the FPGA, making it difficult to further improve the amplification factor, thus restricting the improvement of resolution and measurement accuracy. Conversely, while time interpolation has high measurement accuracy, it requires the introduction of a long delay line when measuring large time intervals, resulting in a significant increase in hardware complexity. Based on the above considerations, this paper combines “coarse counting” and “fine time” measurement to design a TDC, which effectively improves time resolution and overall accuracy while maintaining the measurement range.

#### 2.4.1. Carry Chain TDC Implementation

The time measurement result output by the TDC consists of two parts: one is obtained by the clock counter and is called “coarse time”; the other is measured by the delay line and is called “fine time”, which is used to characterize the amount of time that the clock counter cannot distinguish, which is less than one clock cycle. By interpolating this time window using the delay line, the overall measurement accuracy can be significantly improved.

**Figure 5:**
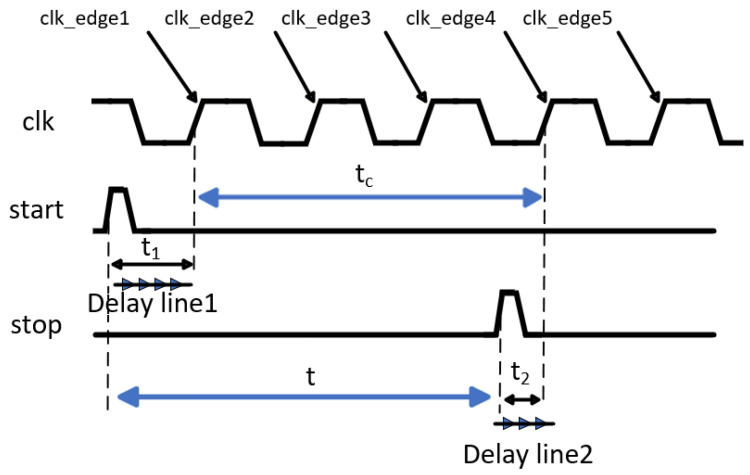
The principle of combining coarse counting with fine time measurement.

Figure 5 illustrates the basic principle of the combined “coarse time-fine time” measurement. Let TDC be used to measure the time interval between the start and stop signals. When the start signal is detected, the clock counter begins counting the rising edges of the clock; when the stop signal is detected, the counting ends. The time interval tc obtained from the counting corresponds to the time between the rising edges clk_edge1 to clk_edge4, the “coarse time”. Simultaneously, the time interval between the start signal and the first subsequent rising edge clk_edge1 is defined as the “fine time” $t_{1}$, measured by interpolation using delay line 1. Similarly, the time interval between the stop signal and the first subsequent rising edge clk_edge4 is the “fine time” $t_{2}$, measured by delay line 2.

Therefore, the time interval Δt between the start signal and the stop signal is

(5) $$t=t_{1}+t_{c}-t_{2}$$

As can be seen from the above measurement principle, the basic idea of time interpolation using delay lines is to subdivide the reference clock into N equal parts using N equally divided delay units, thereby increasing the measurement resolution by N times.

#### 2.4.2. Carry Chain TDC Architecture

**Figure 6:**
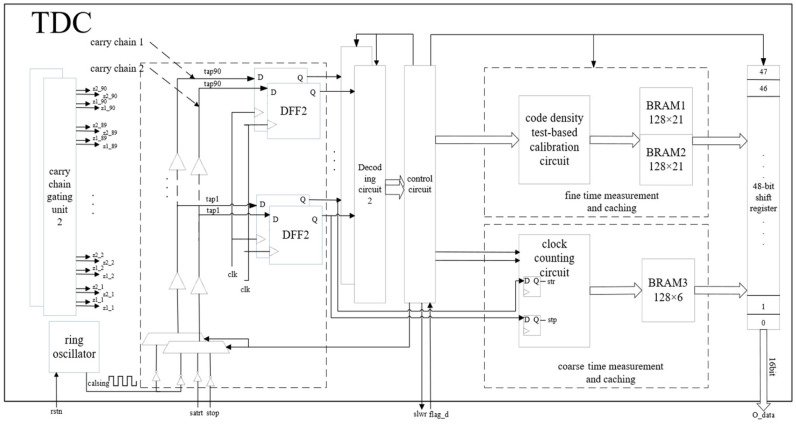
TDC circuit structure.

The overall module includes a carry chain gating unit, a ring oscillator, some delay lines, a decoding circuit, a control circuit, a code density test-based calibration circuit consisting of CAL_RAM and LUT_RAM, a clock counting circuit, and a BRAM storage circuit. Figure 6 shows the TDC circuit structure of this paper. A brief explanation of each functional module follows:

1. The carry chain gating unit keeps the delay lines conducting after the TDC is powered on.
2. The ring oscillator generates a random signal for calibration.
3. Delay line 1 quantizes the time interval between the start signal and the first rising edge of the subsequent clock, while delay line 2 quantizes the time interval between the stop signal and the first rising edge of the subsequent clock. The two quantized time intervals are called “fine time 1” and “fine time 2”, respectively.
4. Since the “fine time” output by the delay lines is thermometer code, it needs to be converted into binary code. Decoding circuits 1 and 2 decoding “fine time 1” and “fine time 2” respectively.
5. The control circuit uses a Finite State Machine (FSM) to control the operating state and data flow of the TDC circuit.
6. The calibration circuit, designed based on the code density testing principle, is implemented using a dual-port RAM ip. The four RAMs are CAL_RAM1, CAL_RAM2, LUT_RAM1, and LUT_RAM2, with corresponding sizes of 256 × 15, 256 × 15, 256 × 21, and 256 × 21, respectively.
7. The clock counting circuit is responsible for counting the clock cycles between the start and stop to obtain the “coarse time”.
8. BRAM1, BRAM2, and BRAM3 are used to store the accumulated values read from LUT_RAM1 and LUT_RAM2, as well as the “coarse time” output by the clock counting circuit, respectively.

#### 2.4.3. Carry Chain TDC Power-On Process

**Figure 7:**
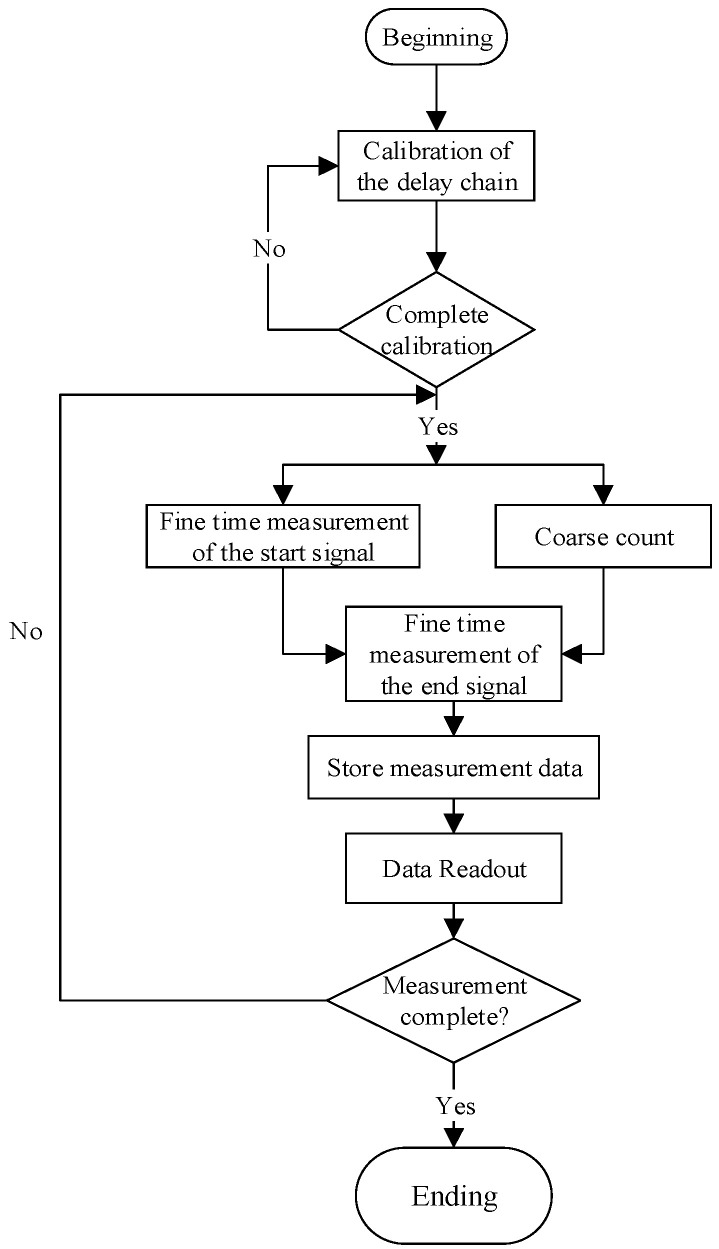
TDC power-up sequence.

Figure 7 shows the TDC power-on workflow, which mainly consists of three steps: delay line calibration, time interval measurement, and data readout.

(1) Delay line calibration

After the TDC is powered on and reset, the TDC first enters calibration mode. The random signal required for calibration is provided by an asynchronous pulse generated by a ring oscillator. It is important to note that the free-running asynchronous signal generated by the ring oscillator for calibration ensures that the measured events are uniformly distributed relative to the TDC reference clock within one clock cycle. Temperature-induced frequency drift only affects the phase ergodicity and does not disrupt the uniformity of the event distribution. This asynchronous pulse is simultaneously fed into the carry-in terminals of two delay lines. The “1/0” transition signal formed on the delay lines is sampled by the system clock, and the sampling result is an output in the form of thermometer code, which is converted into the corresponding binary code in the decoder. Subsequently, the decoded binary code is used as the read/write address of CAL_RAMs. The original data at that address is read, incremented by 1, and written back. After calibration, the value stored at each address in CAL_RAMs reflects the cumulative number of transitions of the corresponding delay unit, which is used for correction in subsequent time measurements.

(2) Time interval measurement

After writing to the LUT_RAMs is complete, the TDC enters the measurement state when the start signal arrives. The start signal first passes through the delay line sampling and decoding circuit 1 to generate the corresponding binary code, which is used as the read address of LUT_RAM1 to read the stored accumulated value and write it to BRAM1. At the same time, the start signal generates the start counting flag, “start_ready”, after being synchronized by two stages of flip-flops, driving the clock counter to start counting clock cycles.

When the stop signal arrives, it also passes through the delay line sampling and decoding circuit 2 to obtain binary code, which is used as the read address of LUT_RAM2. The corresponding accumulated value is read and written to BRAM2. After the stop signal is synchronized by two stages of flip-flops, an end-counting flag, “stop_ready”, is generated. The clock counter then stops counting, outputs the “coarse time”, and writes it to BRAM3.

Given the clock period T and the number of calibrations N, if the accumulated values read from LUT_RAM1 and LUT_RAM2 are $X_{1}$ and $X_{2}$, respectively, the measurement time result can be further calculated.

“Detailed Time” 1 is

(6) $$t_{1}=\frac{X_{1}}{N}T$$

“Detailed Time” 2 is

(7) $$t_{2}=\frac{X_{2}}{N}T$$

The difference between the delay of the start signal from the port to the carry input of delay line 1 and the delay of the stop signal from the port to the carry input of delay line 2 is called the fixed delay calibration value $t_{ca}$. If the “coarse time” is $t_{c}$, then the time interval Δt can now be obtained as follows.

(8) $$t=\frac{X_{1}-X_{2}}{N}T+t_{c}+t_{ca}$$

(3) Data Readout

When BRAM1, BRAM2, and BRAM3 are full, the enable-writing signal is set, and the system enters a data-reading state. In this paper, the “accumulated value 1” and “accumulated value 2” of TDC are both 21 bits wide, and the “coarse time” is 6 bits wide, while the USB bus is only 16 bits wide, which cannot complete all data transmissions in a single clock cycle. Therefore, a 48-bit shift register was designed for data concatenations and outputs.

In data read mode, “fine time 1”, “fine time 2”, and “coarse time” are first read from BRAM1, BRAM2, and BRAM3, respectively, and written parallel to the shift register in descending order of the most significant bit to form a complete data frame. Subsequently, in each clock cycle, the lower 16 bits of the shift register are output to the USB bus, while the register content is shifted right by 16 bits. Once the current data frame is completely output, the next data address is read from the BRAMs; this continues until all data transmissions are complete. The state switching of the TDC and the data interaction between various functional modules are all coordinated by the control circuit.

**Figure 8:**
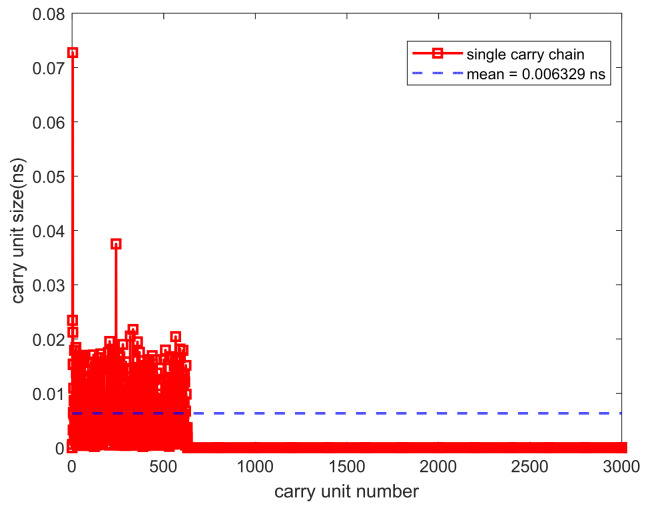
Single-carry chain TDC measurement results.

Figure 8 shows the single-carry chain TDC measurement results. The results fluctuate in the range of 45.4753 ps to 0.432 ps, with an average of 6.329 ps.

### 2.5. Inter-Chain Partitioning and Implementation of Eight-Parallel-Multi-Carry Chain TDC

The non-uniformity of carry chain units and the large delay of a single carry unit are the main factors restricting the measurement accuracy of TDC. To address this issue, this paper proposes an inter-chain segmentation method based on the traditional 8-parallel-multi-carry chain structure. By cross-segmenting and recombining 8 parallel multi-carry chains, an equivalent carry chain is constructed [31]. This equivalent carry chain has a finer equivalent carry unit granularity, thereby achieving a time resolution superior to that of a physical single-carry unit delay.

#### 2.5.1. TDC Inter-Chain Splitting

**Figure 9:**
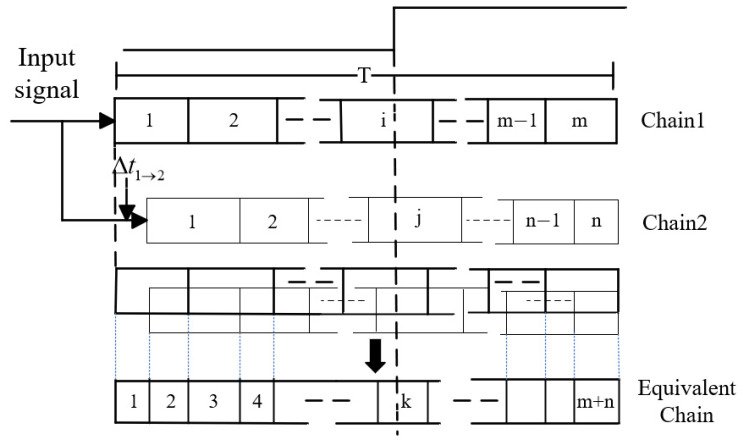
Schematic diagram of inter-chain partitioning method.

Using two carry chains as an example makes the inter-chain partitioning method easier to explain. Figure 9, chain 1 and chain 2 each have m and n (m≈n) carry units, respectively. Different rectangles represent different carry unit sizes, showing that the carry unit sizes are not uniform across the chains and are also inconsistent between the two chains. Furthermore, the physical placement of the different carry chains is also different, resulting in different wiring delays from the signal input port to each carry chain.

Based on the above two points, for the same hit signal, the number of carry units traversed in different carry chains is also different. Figure 9, the trace delay of the second chain relative to the first chain is ${\Delta t}_{1\to 2}$. The hit signal propagates through i carry units in the first carry chain, j carry units in the second carry chain, and k carry units in the equivalent carry chain, where k=i+j. When the two carry chains are split and merged into one equivalent carry chain, the larger carry units are effectively divided. The average resolution of carry chain 1 is $\rho_{1}=T/m$, the average resolution of carry chain 2 is $\rho_{2}=T/n$, and the average resolution of the equivalent carry chain is $\rho_{e}=T/(m+n)\approx \rho_{1}/2\approx \rho_{2}/2$, which is half the average resolution of a single carry chain.

According to the code density calibration method, for a total of N calibration signals, the number of calibration signals falling on the i-th carry unit of the first carry chain is ni, and the number of calibration signals falling on the j-th carry unit of the second carry chain is nj. Therefore, the sizes of the i-th carry unit of carry chain 1 and the j-th carry unit of carry chain 2 are $\mu_{1i}=\;(ni\times T)/N$ and $\mu_{2j}=\;(nj\times T)/N$, respectively. A calibration signal falling on both the i-th carry unit of the first carry chain and the j-th carry unit of the second chain is equivalent to one falling on the k-th carry unit of the equivalent carry chain. Assuming the number of calibration signals that fall on both the i-th carry unit of the first carry chain and the j-th carry unit of the second chain is $\theta_{k}$, the size of the k-th carry unit of the equivalent carry chain can be expressed as

(9) $$\mu_{k}=\frac{\theta_{k}T}{N}$$

The remaining carry units of the equivalent carry chain are calibrated in the same way.

Next, we will discuss how the inter-chain segmentation method improves the uniformity and temperature resistance of the carry chain. When all carry units are uniform, the root mean square error is caused by quantization error and can be written as

(10) $$\sigma^{2}=\frac{\varepsilon^{2}}{12}$$

where ε is the size of a uniform carry unit. When the carry units are inconsistent, the mean square error can be written as

(11) $$\sigma^{2}=\sum_{i}\left(\frac{\varepsilon_{i}^{2}}{12}\times \frac{\varepsilon_{i}}{H}\right)$$

where $\varepsilon_{i}$ is the size of the i-th carry unit in the ordinary carry chain, $H=\sum_{i}\varepsilon_{i}$. The carry unit $\varepsilon_{ei}$ of the equivalent carry chain can be written as

(12) $$\varepsilon_{ei}=c_{i}\times \varepsilon_{i}$$

where 0 < ci ≤ 1; the mean square error can be written as

(13) $$\sigma_{e}^{2}=\sum_{j}\left(\frac{\varepsilon_{ei}^{2}}{12}\times \frac{\varepsilon_{ei}}{H}\right)$$

In Equation (11), the larger the $\varepsilon_{i}$, the greater its contribution to the mean square error $\sigma_{e}^{2}$. After segmentation, the carry units with larger values $\varepsilon_{ei}$ are effectively segmented. Equation (13) shows that the mean square error of the equivalent carry chain becomes smaller, especially when the degradation of the mean square error caused by carry units with larger values is significantly improved.

Regarding the effect of temperature on the size of the carry unit, since the structure of the carry units is the same, it can be assumed that the effect of temperature changes on each carry unit is consistent. Assuming the temperature coefficient of the carry unit is L, the size of the carry unit in the normal carry chain after temperature changes can be written as follows:

(14) $${\varepsilon_{i}}_{ti}=L\times \varepsilon_{i}$$

The carry unit size of the equivalent carry chain can be written as

(15) $${\varepsilon_{i}}_{ti}=L\times c_{i}\times \varepsilon_{i}$$

In Equations (14) and (15), after the temperature changes, the change value of the carry unit in the equivalent carry chain is smaller. This can be more intuitively explained as the change in a single carry unit caused by temperature changes being divided by the other carry units, thus leading to better temperature resistance, which is reflected in the TDC having a lower temperature coefficient.

#### 2.5.2. 8-Parallel-Multi-Carry Chain TDC Implementation

**Figure 10:**
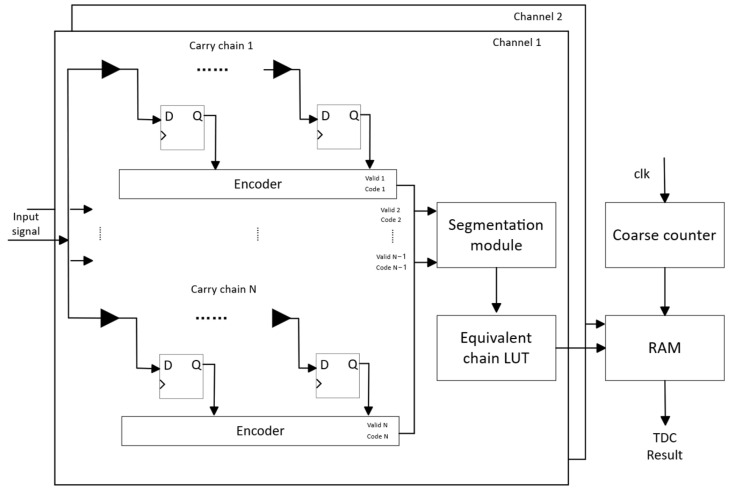
Multi-carry chain TDC block diagram.

A 2-channel TDC based on multi-carry chains cross-segmentation method was implemented on a Xilinx 47DR device. The architecture of the TDC is shown in Figure 10. N is set to 8, implementing a 8-parallel-multi-carry chain TDC, with each channel consisting of 8 carry chains for “fine time” measurement. After encoding, the binary code and valid signal of each chain are sent to the segmentation module. The segmentation module adds the binary codes of each chain to obtain the binary code of the equivalent carry chain, which is stored in RAMs as an equivalent carry chain lookup table, thus achieving calibration of the equivalent carry chain. A counter is used to record the number of clock cycles for “coarse time” measurement. The clock frequency is 400 MHz. The time interval between the two channel signals can be calculated from the “coarse time” and “fine time” stored in RAMs. Each functional module in Figure 10 operates in its respective clock domain, driven by a unified system reference clock.

**Figure 11:**
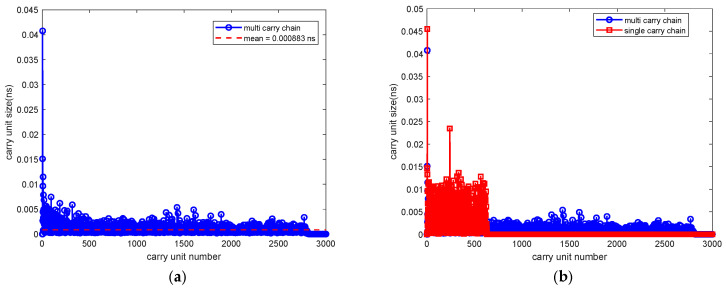
Measurement results: (**a**) 8-parallel-multi-carry chain TDC; (**b**) comparison.

Figure 11 shows the phase measurement results of the 8-parallel-multi-carry chain TDC. The phase measurement accuracy is significantly better than that of the single-carry chain, with a phase measurement error fluctuation ranging from 0.285 ps to 40.754 ps and an overall average of only 0.883 ps. Further comparison with the resolution index of the single-carry chain TDC in Figure 8 reveals a clear eight-fold linear correlation between the resolution of the parallel eight-chain architecture and the single-chain architecture; that is, the resolution of the eight-chain architecture is eight times that of the single-chain architecture. Figure 11a visually demonstrates the significant performance difference in phase measurement accuracy between the two architectures, fully verifying the role of the 8-parallel-multi-carry chain architecture in improving the phase measurement resolution of the TDC.

## 3. Experimental Results and Analysis

### 3.1. Phase Measurement

#### 3.1.1. MMCM Phase-Shift Measurement

**Figure 12:**
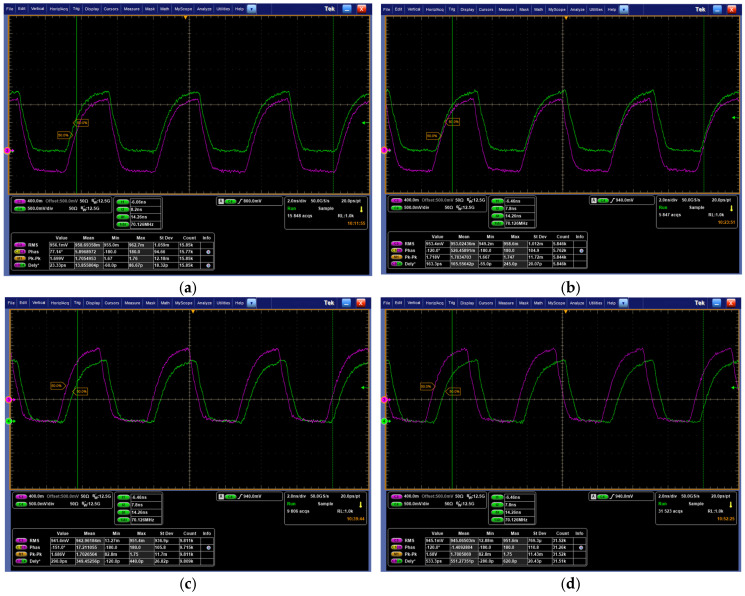
Oscilloscope observations of phase shift: (**a**) Initial non-phase-shifted; (**b**) phase shift 178.571 ps; (**c**) phase shift 357.142 ps; (**d**) phase shift 535.714 ps.

**Figure 13:**
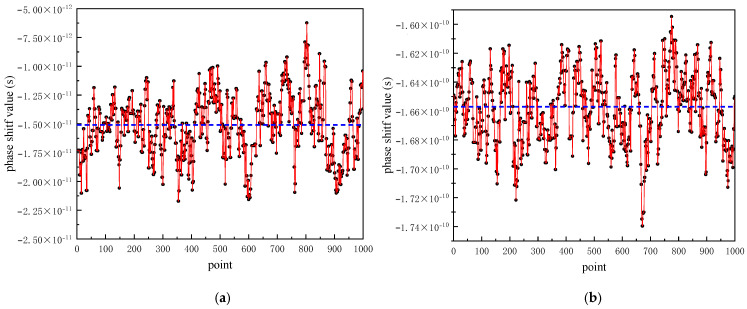

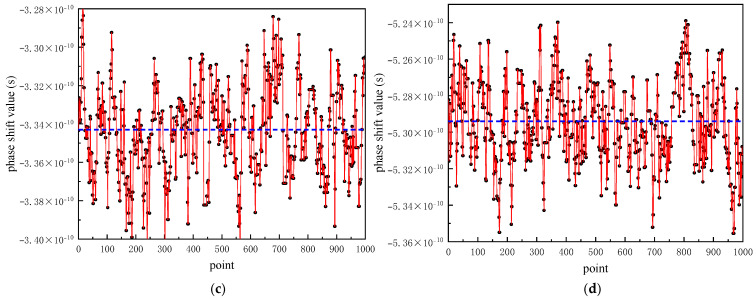
Collect 1000 sample data points: (**a**) Initial non-phase-shifted result; (**b**) phase-shift 178.571 ps result; (**c**) phase-shift 357.142 ps result; (**d**) phase-shift 535.714 ps result. The black dots are the oscilloscope observations of the phase difference sampled 1000 times consecutively. The blue dashed line represents the average of the 1000 phase difference measurements.

First, at room temperature (25 °C), three 200 MHz signals with phase differences of 178.5714 ps, 357.1428 ps, and 535.7142 ps generated by the dynamic phase shift of the MMCM were measured multiple times, as shown in Figure 12. For each phase shift, 1000 sample data points were collected using an oscilloscope and their mean values were calculated, which are plotted in Figure 13 for further analysis. Then, the root mean squares (*RMS*), standard deviations (*σ*), and means of each data set were calculated, as shown in Table 1.

*CTV* (Compensated True Value) can be written as

(16) CTV=RMS+15.4 ps

In practical hardware systems, wiring delays and cable length inconsistencies along different paths lead to a non-negligible fixed delay in the initial non-phase-shifted phase. Table 1 shows that this fixed delay is 15.4 ps. To eliminate its interference with measurement results, all subsequent measurements during the phase-shifting processes must compensate for this fixed delay. Instead of using *RMS* for comparison, we define a new variable *CTV* in Equation (16), the artificially compensated true phase-shift value, and compare it with the theoretical setting points at the top of Table 1. *CTV*s indicate that when the phase-shifting module is working normally, the actual phase-shift value *CTV*s fluctuate within 10 ps above and below the theoretical setting points, with a standard deviation of approximately 2–3 ps.

Next, we will compare their phase measurement accuracy using three methods: DDMTD, single-carry chain, and 8 parallel multi-carry chains.

#### 3.1.2. DDMTD Phase-Shift Measurement

**Figure 14:**
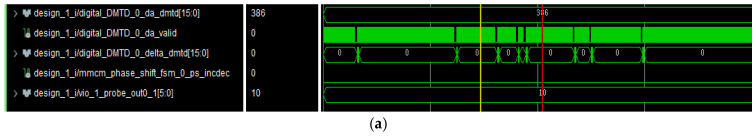

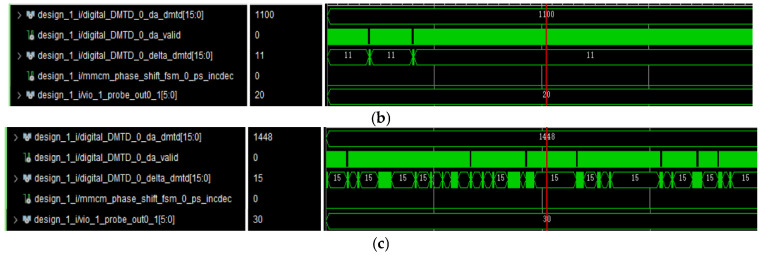
Results by DDMTD method: (**a**) Phase shift 178.571 ps; (**b**) phase shift 357.142 ps; (**c**) phase shift 535.714 ps.

We will use the DDMTD method on the Xilinx 47DR FPGA platform; results are shown in Figure 14.

Figure 14 shows the waveform capture interface of ila ILA in Vivado. da_dmtd is the result of continuously capturing 100 ${delta}_{dmtd}$. So Δt can be written as

(17) $$\Delta t=\frac{da\_dmtd}{100\times clk\_cnt\times N}$$

**Figure 15:**
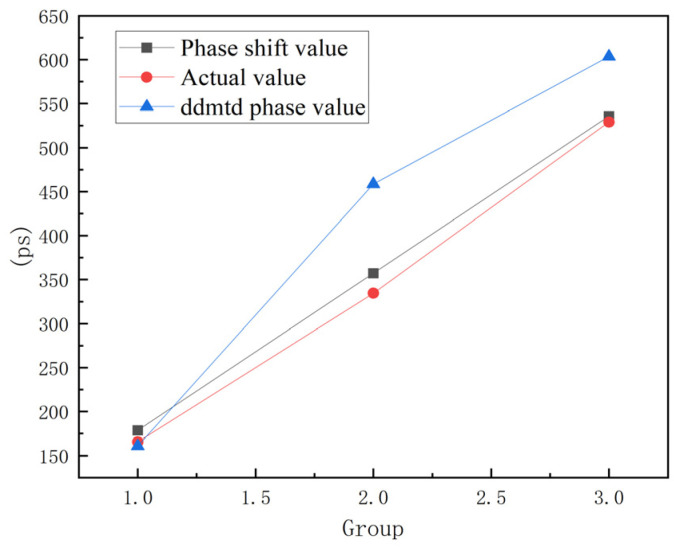
Phase measurement results by DDMTD.

Referring to the analysis in Section 2.3.2, the phase difference results obtained through calculation are shown in Figure 15. The phase measurement error of DDMTD fluctuates in the range of 20 ps to 75 ps.

Next, we used the single-carry chain method and eight-parallel-multi-carry chain method for measurement.

#### 3.1.3. Carry Chain Phase-Shift Measurement

**Figure 16:**
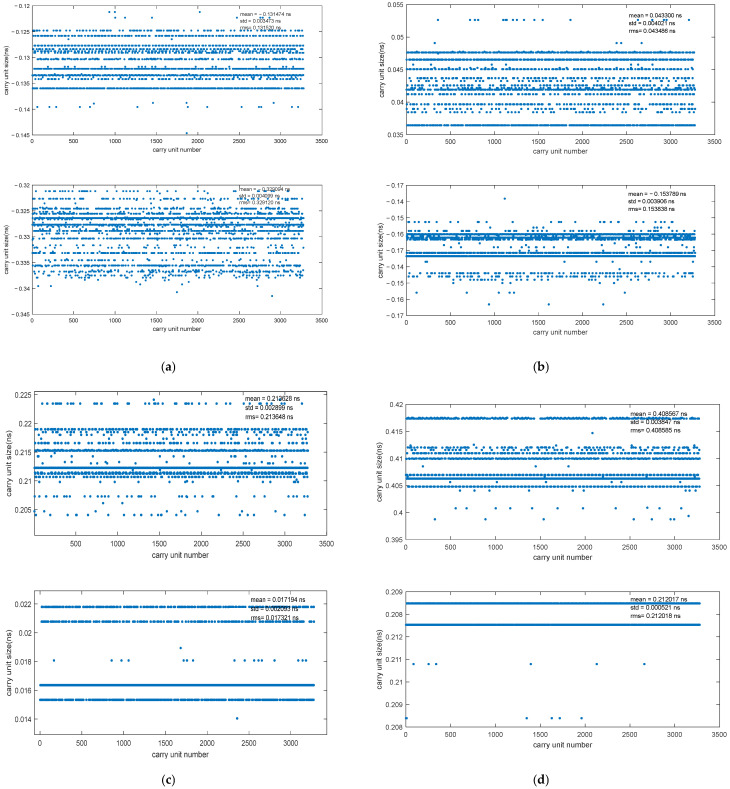
Results by carry chain methods: (**a**) Initial non-phase-shifted; (**b**) phase shift 178.571 ps; (**c**) phase shift 357.142 ps; (**d**) phase shift 535.714 ps.

Results of the single-carry chain and eight-parallel-multi-carry chain methods are shown in Figure 16.

Due to differences in FPGA internal layouts and routings, the actual delays of the carry chain inherently exist on the circuit board. This delay is a critical error source for the system and cannot be ignored; it must be eliminated through compensation. In Figure 16a, before measuring, the single-carry chain TDC and the eight-parallel-multi-carry chain TDC already have inherent offsets of 131.52 ps and 329.12 ps, respectively. Therefore, compensation is required before the formal phase measurement begins. We define new variables CMV1 and CMV2, which are written as Equations (1) and (2), as compensation values for the single carry chain and eight parallel multi-carry chains, respectively, for subsequent comparison with theoretical values. Based on this basis, inherent offset compensation is performed on the original measurement results in Figure 16b–d.

(18) CMV=RMS(i)+131.52 ps

The 8-parallel-multi-carry chain compensation can be written as Equation (19).

(19) CMV=RMS(i)+329.12 ps

In the formula, RMS(i) represents the root mean square value of the i-th measurement, with the same sign as the mean value of the i-th measurement; CMV1 and CMV2 are 174.96 ps and 175.282 ps, 345.122 ps and 346.441 ps, and 540.059 ps and 541.138 ps, respectively. The phase measurement errors for single-carry chain and 8-parallel-multi-carry chain measurements are obtained by subtracting CMV from CTV, which are −6.04 ps and −5.718 ps, −4.778 ps and −3.459 ps, and −4.541 ps and −3.462 ps, respectively.

Comparative experiments at room temperature demonstrate that the 8-parallel-multi-carry chain TDC exhibits the best phase measurement accuracy. Specifically, although the single-carry chain TDC shows improved measurement accuracy after code density calibration, and its overall accuracy is significantly better than the DDMTD method (Figure 16), the eight-parallel-multi-carry chain architecture, based on the core design of inter-chain segmentation, significantly improves phase measurement resolution while optimizing phase measurement accuracy. Its key indicators, *RMS* and STD, are significantly better than the traditional single-carry chain TDC, fully validating the technical advantages and feasibility of the eight parallel multi-carry chains’ segmented architecture.

### 3.2. Temperature Drift

To systematically evaluate temperature drift characteristics and verify whether the inter-chain partitioning architecture can significantly reduce the impact of temperature changes, a thermostat was used to heat the FPGA chip. Multiple repeated measurements were performed at different temperature settings (10 °C to 100 °C) to observe the LSB value changes in single-carry chain TDC and eight-parallel-multi-carry chain TDC. Considering that the implementation of the DDMTD module mainly relies on a fixed programming algorithm and does not utilize onboard hard-core resources. The algorithm accuracy is theoretically unaffected by ambient temperature; therefore, temperature drift characteristic analysis is only applicable to carry chain methods.

**Figure 17:**
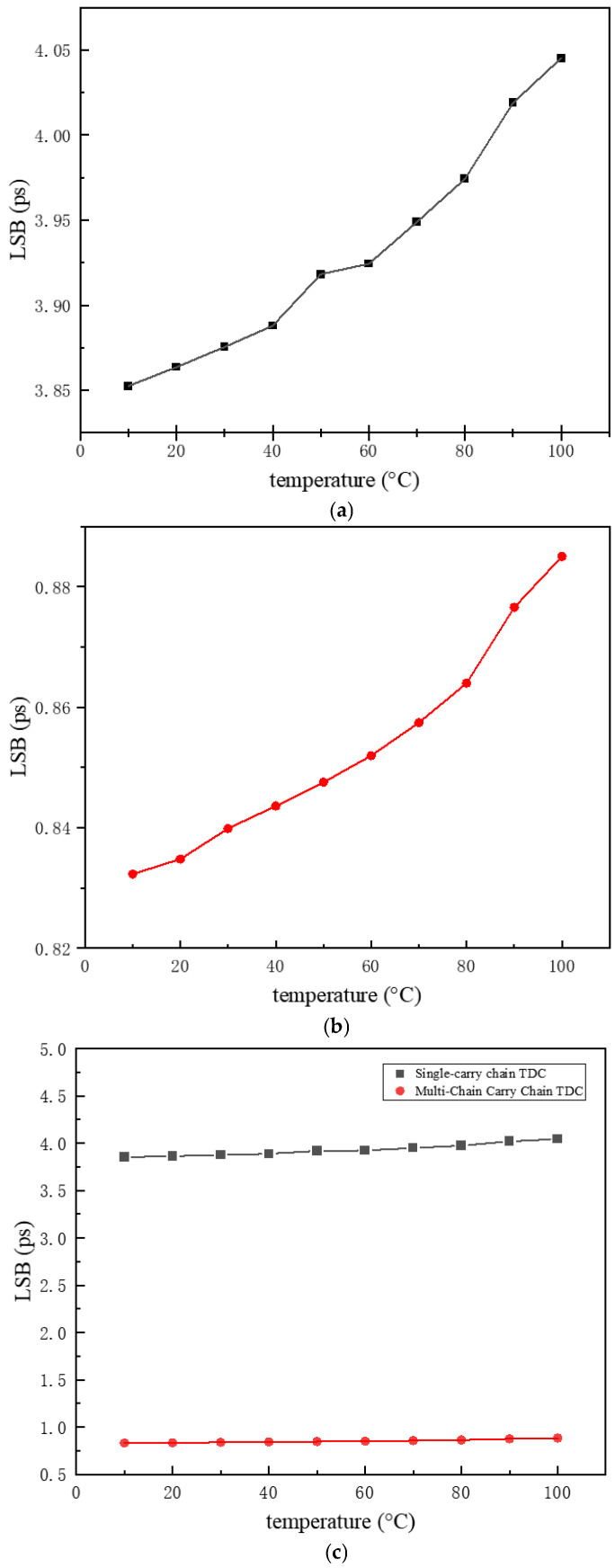
LSB of the TDC with different chain numbers varying with ambient temperature: (**a**) Chain number is 1; (**b**) chain number is 8; (**c**) combined results plot.

In Figure 17, we observed that the LSB of the single-carry chain TDC exhibits an almost linear relationship with temperature. When the temperature increases from 10 °C to 100 °C, the LSB increases from 3.85 ps to 4.05 ps, with a temperature coefficient of 0.002127 ps/°C. In contrast, the 8-parallel-multi-carry chain TDC using the inter-chain segmentation method remains almost unchanged at different temperatures, with the LSB increasing only from 0.833 ps to 0.885 ps, and a temperature coefficient as low as 0.000564 ps/°C. These results demonstrate that the 8-parallel-multi-carry chain TDC using the inter-chain segmentation method has extremely low temperature coefficients and can effectively reduce the impact of temperature changes.

### 3.3. Nonlinearity

**Figure 18:**
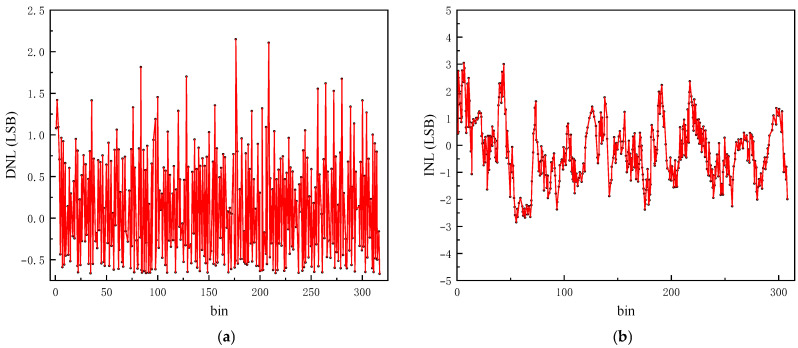
Nonlinearity of the TDC with 8 chains: (**a**) differential nonlinearity, (**b**) integral nonlinearity.

Differential nonlinearity (DNL) and integral nonlinearity (INL) are commonly used to evaluate the nonlinear performance of TDC. DNL describes the deviation of each actual bin width from the ideal average bin width, while INL represents the cumulative sum of DNL from the first bin to a given bin, which can be derived from the measured bin width data. Figure 18a,b show the DNL and INL results for the 8-parallel-multi-carry chain TDC, respectively. The measured DNL ranges from −0.99 LSB to 4.79 LSB, while the INL ranges from −14.18 LSB to 13.16 LSB.

### 3.4. Resource Usage and Power Consumption

Table 2 shows the resource usage and power consumption of the single-carry chain and the 8-parallel-multi-carry chain TDC. The main resources used include look-up tables (LUTs), flip-flop registers (FFs), blocks of random access memory (BRAMs), and phase-locked loops (PLLs). We can discover that the 8-parallel-multi-carry chain TDC consumes more resources than the single-carry chain TDC and the DDMTD.

## 4. Discussion

We focus on high-precision time synchronization measurement, implementing three methods for phase measurement on the Xilinx 47DR FPGA platform: the single-carry chain TDC, the 8-parallel-multi-carry chain TDC with code density calibration and inter-chain segmentations, and the DDMTD. Experiments used an MMCM dynamic phase-shift module to generate three picosecond-level phase difference signals, with theoretical values of 178.5714 ps, 357.1428 ps, and 535.7142 ps, respectively. A high-precision oscilloscope was used to obtain comparative reference values, providing a reliable reference for subsequent accuracy evaluation. Experimental results show that, in terms of measurement accuracy, the 8-parallel-multi-carry chain TDC achieves a root mean square error of less than 1 ps and a temperature coefficient of 0.000564 ps/°C within a temperature range of 10–100 °C. Its performance is better than the 3.85 ps of the single-carry chain method and far superior to the 20–75 ps of the DDMTD method. In terms of hardware implementation, the DDMTD method has the lowest power consumption and resource consumption, but its resolution and phase measurement accuracy are correspondingly poor. A summary table of key indicators (resolution, accuracy, and temperature coefficient) for the three phase measurement methods has been drawn in this paper, as shown in Table 3.

In summary, FPGA-based 8-parallel-multi-carry chain TDC exhibits significant advantages in picosecond-level time synchronization measurement resolution, while the DDMTD method is more suitable for applications requiring high long-term stability and low frequency drift. Due to their complementary structural and performance characteristics, these two methods provide valuable references for designing time synchronization systems that combine high accuracy and high stability. Bin-by-bin calibration architecture proposed in this paper is not only applicable to the experimental scenario studied in this paper, but also demonstrates good versatility and scalability for general-purpose timing and time measurement systems based on FPGAs.

Future work will not only focus on further improving the adaptive calibration mechanism and error compensation model, but also on introducing advanced optimization and learning-based calibration techniques to enhance the robustness of the system under PVT variations. In particular, the Temperature-Friendly Gradient Descent (TFGD) method is considered a practical and hardware-efficient approach for enabling online adaptive calibration in FPGA-based TDC systems. The core idea of TFGD is to continuously update the calibration LUT_RAM according to real-time timing error feedback observed during normal operation, allowing the quantization mapping to gradually adapt to slow environmental changes. Compared with conventional static code density calibration methods relying on offline statistics, this strategy is inherently more suitable for long-term deployment, as it mitigates calibration aging and performance degradation caused by PVT drift. From an optimization perspective, TFGD can be regarded as an engineering-oriented and implementation-friendly form of gradient-based iterative adjustment, where calibration parameters are updated using lightweight arithmetic operations and limited memory access. This makes it particularly compatible with FPGA platforms, in which the high-speed measurement data path is handled by the programmable logic, while a soft-core processor or processing system performs background calibration updates without disturbing real-time TDC operation. To further strengthen the theoretical foundation of this approach, recent advances in tempered fractional gradient descent and fractional-order optimization provide a compelling and rigorous framework. Unlike conventional integer-order gradient descent, fractional gradient methods introduce a memory-aware update mechanism, in which historical gradient information contributes to the current parameter update through a power-law or tempered kernel. This characteristic has been shown to significantly improve robustness, convergence stability, and noise tolerance in non-stationary and stochastic optimization problems, which are increasingly studied in the robust learning and adaptive control literature. More recent tempered fractional gradient descent formulations further incorporate exponential tempering factors, effectively limiting the long-range memory effect while preserving the benefits of fractional-order dynamics. This tempering mechanism enables a favorable balance between adaptability and stability, preventing excessive sensitivity to outdated information and ensuring bounded computational complexity. Such properties are particularly attractive for time-critical and resource-constrained systems, where strict real-time constraints coexist with slowly varying system parameters. In the context of FPGA-based TDC calibration, PVT-induced timing errors exhibit exactly these characteristics: slow drift, non-stationarity, nonlinear accumulation, and partial temporal correlation. Within this framework, the proposed TFGD strategy can be interpreted as a hardware-friendly approximation of tempered fractional-order optimization, where historical timing error information is implicitly accumulated through continuous LUT updates rather than explicitly stored high-order gradients. This interpretation elevates the calibration approach from a heuristic rule to a principled optimization strategy grounded in modern robust learning theory. With appropriate timing isolation and double-buffering mechanisms, the online calibration process can be executed in a non-blocking manner, enabling true real-time adaptive calibration without interrupting normal TDC measurements. This strategy is expected to significantly enhance long-term stability and self-recovery capability under complex environmental variations.

In addition, future research may further explore extended parallel multi-carry chains TDC architecture combined with delay equalization and co-calibration strategies, aiming to achieve higher consistency and lower noise performance in large-scale parallel time measurement systems. Through these improvements, FPGA-based high-precision time synchronization and measurement technologies are expected to demonstrate broader application potential in satellite navigation, distributed radar systems, and high-speed communication networks.

## Figures and Tables

**Table 1:** MEAN, *RMS*, STD, and *CTV* at different phase-shift values.

Performance Metrics	Unshifted Phase	Phase Shift 178.5714 ps	Phase Shift 357.1428 ps	Phase Shift 535.7142 ps
MEANS (ps)	−15.1	165.7	334.3	529.4
*RMS* (ps)	15.4	165.6	334.5	529.2
STD (ps)	2.7	2.4	2.3	2.2
*CTV* (ps)	/	181	349.9	544.6

**Table 2:** Resource usage and power consumption of the TDC.

Resource	Available	1-Chain		5-Chain		DDMTD	
		Use	Use (%)	Use	Use (%)	Use	Use (%)
LUT	425,280	3558	0.84	12,718	2.99	2026	0.48
LUTRAM	213,600	266	0.12	199	0.09	192	0.09
FF	850,560	6941	0.82	30,196	3.55	3300	0.39
BRAM	1080	89	8.24	264	24.44	6	0.56
BUFG	696	6	0.86	6	0.86	9	1.29
MMCM	8	1	12.5	1	12.5	2	25.00

**Table 3:** Comparison of three methods.

Method	Resolution (ps)	*CTV* 181 ps Measurement (ps)	*CTV* 349.9 ps Measurement (ps)	*CTV* 544.6 ps Measurement (ps)	Temperature Coefficient (ps/°C)
DDMTD	52.088	160.846	458.371	603.382	/
Single-carry chain	6.329	174.960	345.122	540.059	0.002127
8 Multi-carry chains	0.883	175.282	346.441	541.138	0.000564

## Data Availability

The original contributions presented in this study are included in the article. Please contact the corresponding author if you have any questions.
